# Influence of Different Types of Peroxides on the Long-Chain Branching of PP via Reactive Extrusion

**DOI:** 10.3390/polym12040886

**Published:** 2020-04-11

**Authors:** Sascha Stanic, Gergö Gottlieb, Thomas Koch, Lukas Göpperl, Klaus Schmid, Simone Knaus, Vasiliki-Maria Archodoulaki

**Affiliations:** 1 Institute of Material Science and Technology, TU Wien, Getreidemarkt 9, 1060 Vienna, Austria; e1644135@student.tuwien.ac.at (G.G.); thomas.koch@tuwien.ac.at (T.K.); vasiliki-maria.archodoulaki@tuwien.ac.at (V.-M.A.); 2 Institute of Chemical Technology of Organic Materials, Johannes Kepler University Linz, Altenberger Straße 69, 4040 Linz, Austria; lukas.goepperl@jku.at; 3 Pergan GmbH, Schlavenhorst 71, 46395 Bocholt, Germany; dr.schmid@pergan.com; 4 Institute of Applied Synthetic Chemistry, TU Wien, Getreidemarkt 9, 1060 Vienna, Austria; simone.knaus@tuwien.ac.at

**Keywords:** polypropylene, reactive extrusion, peroxide, long-chain branching, strain hardening

## Abstract

Long-chain branching (LCB) is known as a suitable method to increase the melt strength behavior of linear polypropylene (PP), which is a fundamental weakness of this material. This enables the modification of various properties of PP, which can then be used—in the case of PP recyclates—as a practical “upcycling” method. In this study, the effect of five different peroxides and their effectiveness in building LCB as well as the obtained mechanical properties were studied. A single screw extruder at different temperatures (180 and 240 °C) was used, and long-chain branched polypropylene (PP-LCB) was prepared via reactive extrusion by directly mixing the peroxides. The peroxides used were dimyristyl peroxydicarbonate (PODIC C126), tert-butylperoxy isopropylcarbonate (BIC), tert-Butylperoxy 2-ethylhexyl carbonate (BEC), tert-amylperoxy 2-ethylhexylcarbonate (AEC), and dilauroyl peroxide (LP), all with a concentration of 20 mmol/kg. The influence of the temperature on the competitive prevalent reactions of degradation and branching was documented via melt mass-flow rate (MFR), rheology measurements, and gel permeation chromatography (GPC). However, via extensional rheology, strain hardening could be observed in all cases and the mechanical properties could be maintained or even improved. Particularly, PODIC C126 and LP signaled a promising possibility for LCB in this study.

## 1. Introduction

In 2018, the global production of plastic amounted to 359 million tons, in other words an increase in the order of 3% compared to 2017. Various plastic products are used in a wide range of different applications. The three biggest markets of plastic products are packaging (39.9%), building and construction (19.8%), and automotive (9.9%). In these three areas, polypropylene (PP) is strongly represented, for example, in sectors such as food packaging, hinged caps, microwave containers, pipes, automotive parts, bank notes, etc. Taking into consideration the separation of low-density polyethylene (PE-LD) and high-density polyethylene (PE-HD) into two independent groups, PP (with an amount of 19.3%) was the most relevant polymer for the production of plastic products in 2018 [1]. 

PP’s significance is due to desirable characteristics, such as excellent chemical resistance, high melting point, high tensile modulus of elasticity, high stiffness, low density, and low cost. It also shows excellent flexural fatigue resistance, can be stabilized to provide good thermal aging stability, and is easy processed by injection molding, extrusion, and spinning [2,3]. Nevertheless, linear, i.e. unmodified, PP is challenging in processes like thermoforming, film blowing, blow molding, extrusion coating, and foaming, because of the lack of melt strength and strain hardening [4].

**Figure 1:**
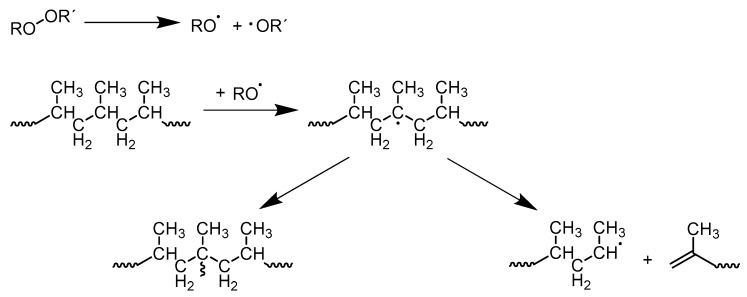
Reaction scheme of long-chain branching (LCB) confronted with β-scission of polypropylene (PP).

Over the past few years, different basic concepts have been studied in the literature to achieve high melt strength PP (PP-HMS) using different methods [5,6,7,8,9]. According to Chikalikar et al. [10], it is possible to increase the melt strength of PP in different ways:

- Increasing the average molecular weight of PP
- Broadening the molecular weight distribution by incorporating high and low molecular weight chain fractions
- Blending PP with polymers such as PE-LD
- Introducing long-chain branching (LCB) on the backbone of PP, which is the most popular method to obtain PP-HMS
- PP-LCB can be achieved in different ways, but reactive extrusion is the most popular process. This type of process offers very short reaction times, little or no use of solvents, simple product isolation steps, and relatively low infrastructure costs [11,12]. In the literature, PP-LCB is a well-studied topic with different approaches to improve the properties of linear PP and their disadvantages [13,14,15,16,17,18,19,20,21,22]. In addition to introducing LCB onto the backbone of PP, further competition reactions can take place. PP in particular tends to undergo chain scission reactions, so-called β-scission, which creates smaller unsaturated fragments of chains [23,24]. There is a possibility that cross-linking occurs, but a prediction of this phenomenon is very difficult. The phenomenon of β-scission caused by the use of peroxides is also known as controlled rheology PP (PP-CR). This leads to a reduction in viscosity, which results in a reduction in average molar mass and a narrowing of PP’s molar mass distribution (MMD) [25]. Additional side reactions could occur, such as disproportionation or a combination of free peroxide radicals. In particular, at higher temperatures, the equilibrium shifts to β-scission or disproportionation, which negatively affects the branching [24]. For this reason, the extrusion temperatures in scientific works are mainly at lower temperatures (180 °C). However, in practice, high temperatures have been used for the extrusion process, which means that additional effects may have occurred such as increased chain scission or different reaction rates during the extrusion. Figure 1 shows a schematic representation of the LCB reaction process confronted with the β-scission of PP. In the first step, the peroxide decomposes under homolytic scission into two primary radicals, which abstract a hydrogen atom from the PP backbone and therefore a PP macroradical is generated. There are now two possible reactions that can take place—on the one hand, the LCB, and on the other hand, the β-scission of PP [26,27].

Lagendijk et al. [28] and Gotsis et al. [29] demonstrated that reactive extrusion using peroxydicarbonate (PODIC), a special class of organic peroxides with different lengths of aliphatic side chains, is a promising method to generate PP-LCB. In recent studies undertaken by our group, we demonstrated that PODIC could be used for upcycling linear isotactic PP with different molar masses and also that the properties of PP with impurities of PE could be improved successfully [30,31,32].

The LCB of PP via reactive extrusion depends on a number of various aspects such as mixing efficiency, shear rate, temperature, and residence time. One of the most important aspects is the choice of the peroxide, which has the largest impact on the reaction. Many attributes need to be addressed [12]. One of the most important attributes of peroxides is the initiator half-time, which is related to their efficiency. Furthermore, the maximum storage temperature is very important and presents a huge challenge to the storage facilities. The peroxide’s primary requirement is the ability to abstract a H atom from the backbone of PP, making it possible to generate PP-LCB. This behavior has been investigated since peroxides play an important role in the modification of PP [33,34]. Klenk et al. [35] reported that peroxides can be classified into seven different types depending on their reactivity based on their chemical structure, namely diacyl peroxides, peroxyesters, diperoxyketals, dialkyl peroxides, hydroperoxides, ketoneperoxides, and peroxydicarbonates. Additionally, Takamura et al. [36] described the effectiveness of peroxides in cross-linking processes depending on their decomposition rates. Three groups of peroxides, that is, group I (diacyl peroxides), group II (peroxyesters), and group III (dialkyl peroxides), were investigated and showed an increase in the lifetime of the peroxides from group I to group III. The choice of the right peroxide plays an important role in generating PP-LCB and the associated opportunity to improve the properties of isotactic PP [27,37,38,39]. Therefore, the influence of five different peroxides and their long-chain branching behavior in conjunction with isotactic PP will is the topic of this study. In order to prove long-chain branching of PP and the associated property changes, the mechanical and rheological behaviors of the samples were investigated. 

## 2. Materials and Methods 

### 2.1. Materials

**Figure 2:**
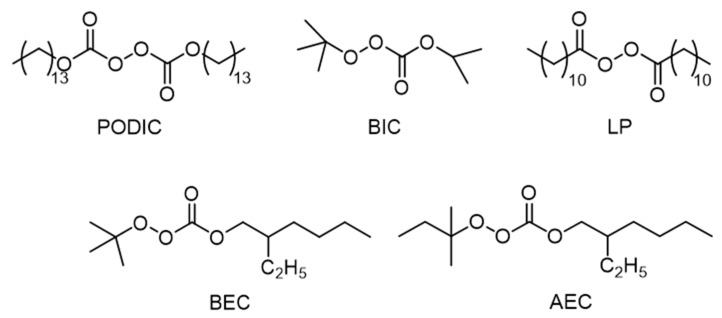
Schematic structure of the peroxides.

Five different peroxides supplied by Pergan GmbH (Bocholt, Germany) were used for the study, and the structure of each peroxide is shown in Figure 2. Differences can be seen in the structure of the peroxides and their decomposition products. The peroxides PODIC C126 (dimyristyl peroxydicarbonate; Pergan GmbH, Bocholt, Germany) and LP (dilauroyl peroxide; Pergan GmbH, Bocholt, Germany) provide two symmetrical reactive units. In comparison, the decomposition products of the peroxides tert-butylperoxy isopropyl carbonate (BIC; Pergan GmbH, Bocholt, Germany), tert-butylperoxy 2-ethylhexylcarbonate (BEC; Pergan GmbH, Bocholt, Germany), and tert-amylperoxy 2-ethylhexylcarbonate (AEC; Pergan GmbH, Bocholt, Germany) result in two unsymmetrical units.

PODIC C126 (Peroxan C126, flakes) is used for the (co)polymerization of vinyl chloride and vinylidene chloride. BIC (Peroxan BIC, solution in odorless white spirits) is a peroxyester for the (co)polymerization of styrene, acrylates, and methacrylates and for the curing of unsaturated polyester resins. In addition, BEC (Peroxan BEC, liquid) and AEC (Peroxan AEC, liquid) are counted among the group of peroxyesters and both are also used for the (co)polymerization of styrene, acrylates, and methacrylates. LP (Peroxan LP, flakes) is an example for the diacylperoxide group and is used for the curing of highly filled methacrylic resins, typically with other peroxides of different reactivity. The characteristics of the different peroxides are summarized in Table 1.

Isotactic PP homopolymer with a melt mass-flow rate (MFR) of 2.8 g/10min was supplied by Borealis (HC 600TF, Vienna, Austria). This is a PP grade for different thermoforming applications. 

### 2.2. Sample Preparation

The sample preparation i.e., the reactive extrusion, was done in an Extron EX-18-26-1.5 single screw extruder (Extron Engineering, Ltd, Akaa, Finland) with an length/diameter (L/D) ratio of 25:1 and 3 independent heating zones (feeding zone, extrusion zone, and die zone). The screw had the following dimensions: screw diameter of 18 mm, channel depth of 1 mm, pitch length of 14 mm, and flight width of 2.5 mm. The reactive extrusion was carried out with a screw speed of 70 rpm at 180 (165, 180, and 220 °C) and 240 °C (165, 240, and 240 °C) to simulate industry-related requirements. According to Giles et al. [40], the calculation of the shear rate ($\dot{\gamma }$) in an extruder screw is done by using Equation (1), where D is the screw diameter in mm, N is the screw speed in rpm and h is the channel depth in mm. Therefore, a shear rate of 66 s^−1^ in the screw channel of the extruder was calculated according to the above-mentioned parameters.

(1) $$\dot{\gamma }=(\pi \times D\times N)/(60\times h)$$

The different peroxides were directly mixed with the PP granulate and added to the extruder. All formulations had the same peroxide concentration (20 mmol) with respect to their molar mass, whereby the various contents of percent by weight (wt %) occur. Sample names and compositions are given in Table 2.

For further sample preparation, the modified PP compositions were shredded with a universal cutting mill “Pulverisette 19” (Fritsch, Idar-Oberstein, Germany) with a 4 mm square perforation sieve and a high-performance cyclone to achieve a better separation.

### 2.3. Characterization of Molar Mass and Viscosity

The characterization of the molar mass and the viscosity of the different samples was done by using dynamic rheology measurements, extensional rheology, high-temperature gel permeation chromatography (HT-GPC), and MFR measurements.

For the dynamic rheology, i.e., the frequency sweep, measurement discs with a diameter of 25 mm and a thickness of 1.2 mm were compression-molded at 180 °C or 240 °C and a pressure of 30 bar. The dynamic rheology was done on a plate–plate system using an Anton Paar MCR 301 rheometer (Graz, Austria), which was equipped with a CTD 450 heating chamber (Anton Paar, Graz, Austria) under nitrogen at 230 °C. This temperature was selected to obtain a better correlation between the dynamic rheology results and the MFR measurements, which used the same temperature. The gap size was 1 mm, the chosen frequency range was between 628 and 0.01 rad/s, and the deformation during measurement was raised logarithmically from 1% to 2%.

For extensional rheology, 8 mm wide stripes were cut from sheets with dimensions of 60 mm × 60 mm × 0.8 mm, which were produced by compression molding at 180 °C or 240 °C. A Sentmanat Extensional Rheometer (SER-HPV 1, Xpansion instruments, Tallmadge, OH, USA) for Anton Paar rheometers was used at 180 °C and with three different strain rates ($\dot{\varepsilon }$ = 5 s^−1^; 1 s^−1^; 0.1 s^−1^). The steady shear experiments for the start-up curves were done with a plate–plate system at 180 °C and two different shear rates (0.001 s^−1^ and 0.1 s^−1^).

The molar mass distribution (MMD) was determined on a high-temperature size exclusion chromatographer (HT-SEC) from Polymer Char (Valencia, Spain). The measurements were carried out at 160 °C with 1,2,4-trichlorbenzene as eluent and, for the detection, an infrared detector (IR5-Detector, Polymer Char) was used.

The measurements of the MFR at 230 °C and under 2.16 kg weight were done according to the DIN EN ISO 1133 method A, in g/10min, with a MeltFloW basic (Karg Industrietechnik, Krailling, Germany).

### 2.4. Mechanical Testing—Tensile Test and Tensile Impact Strength

According to ISO 527-2-A5 the test specimens for tensile test were performed by injection molding with a Haake Mini Lab II (Thermo Fisher Scientific, Waltham, MA, USA). The temperature of the twin-screw extruder was selected between 180 °C and 240 °C. The injection molding parameters were set with a 90 °C mold temperature and an injection pressure of 350 bar. The test machine (Zwick 050, ZwickRoell GmbH & Co. KG, Ulm, Germany) was used with a test speed of 10 mm/min and was equipped with a 1 kN load cell and an extensometer.

The impact tensile test specimens (60 mm × 10 mm × 1 mm) were prepared by injection molding under the same conditions. The test specimens were notched on both sides and tested according to ISO 8256/1A on an Instron Ceast 9050 (2 J hammer, crosshead mass = 15 g; Darmstadt, Germany).

## 3. Results and Discussion

### 3.1. Melt Flow Properties

Lagendijk et al. [28] reported that the branched PP, which was modified with various structures of PODIC via reactive extrusion, showed a slightly lower MFR compared to that of the linear PP. Generally, the molecular structures of polymers affect the MFR at low shear rates. To determine the shear rate of the MFR equipment’s die, the following Equation (2) can be used [41,42]. In this study, the virgin PP with an MFR of 2.8 g/10min has a shear rate value of approximately 5 s^−1^.

(2) $$\dot{\gamma }=1.85\times \mathrm{MFR}$$

**Figure 3:**
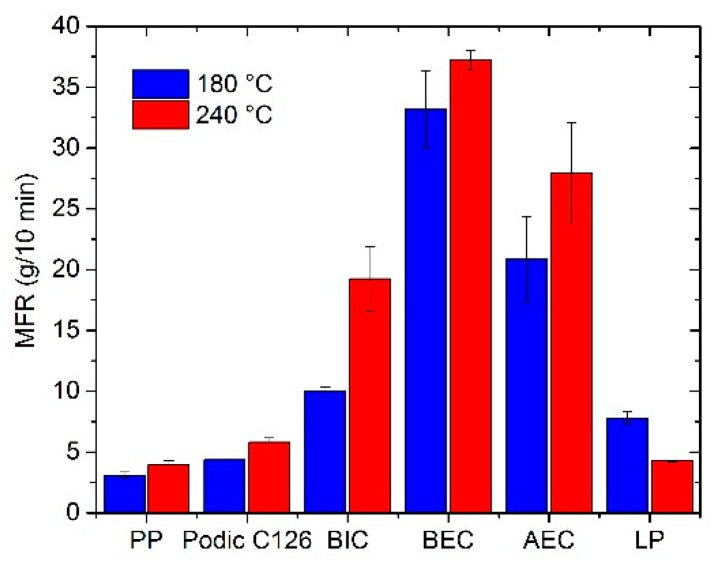
Melt mass-flow rate (MFR) of extruded PP (without peroxide) and modified PP (with different peroxides) at 180 °C and 240 °C; virgin PP has an MFR of 2.8 g/10 min.

On the one hand, a high MFR value is the result of chain mobility because of the β-scission and, on the other hand, long-chain branching or cross-linking reduces the MFR values [27,43]. The MFRs of the unmodified and modified PP for this study under different extrusion temperatures are shown in Figure 3. It can be seen that the MFRs increase to high values for the unsymmetrical peroxides BIC, BEC, and AEC at 180 °C and 240 °C. In this case, the degradation and the associated β-scission of the PP chain are predominant. On the one hand, the peroxides are responsible for the scission; however, the temperature of the reactive extrusion is another aspect of the chain scission reaction, which is very important. The virgin PP that was extruded shows a slightly increasing MFR value in comparison to the virgin granulate with an MFR of 2.8 g/10 min. The symmetrical peroxides PODIC and LP show values minimally higher compared to that of the extruded PP. The modification with PODIC presents the lowest MFR value after extrusion at 180 °C and with LP presents the lowest MFR value at the 240 °C extrusion temperature. However, the modification with LP shows an unexpected behavior and does not follow the trend compared to that of the other peroxides. In all other cases, including the unmodified PP, the MFR values are higher at a modification temperature of 240 °C compared to 180 °C. This suggests that the temperature during extrusion plays an important role in the modification and branching of PP. The low MFR values for the PODIC and LP modifications is the first sign of the highest branching efficiency of these two types of peroxides in comparison to the unsymmetrical ones.

### 3.2. Dynamic Rheology

**Figure 4:**
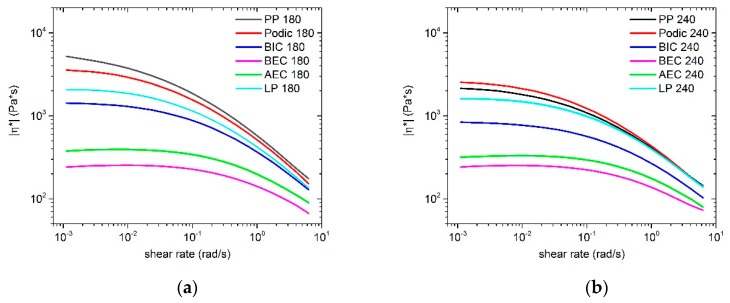
Complex viscosity versus shear rate of samples extruded at (**a**) 180 °C; (**b**) 240 °C; measured at 230 °C.

In the literature, linear viscoelastic rheology is a very well established technique for the detection of LCB, due to the high sensitivity of the LCB structure and with its associated structure change of molecular chains [44,45,46,47,48]. The complex viscosity (η*) is very sensitive to the LCB structure and the peroxide-induced degradation of PP. On the one hand, the presence of very low LCB can change the zero-shear viscosity (η_0_) and the shear thinning degree of modified PP [49]. On the other hand, the peroxide-induced degradation of PP and the resulting β-scission can also change these behaviors [25]. For a better comparison to the MFR values, the complex viscosity versus angle shear rate for all samples measured at 230 °C are shown in Figure 4. The samples reactively extruded at 180 °C are presented in Figure 4a, whereas those modified at 240 °C are presented in Figure 4b. 

For the modification at 180 °C, the curves of all modified PP samples are located below the curve of the virgin PP. In the presence of the various peroxides, the complex viscosity decreased obviously at low frequency and the Newtonian zone became broader, caused by the chain scission during the peroxide-induced reactive extrusion process. However, PODIC and LP show approximately the same behavior as the virgin PP. In particular, PODIC and LP have a higher shear thinning behavior at higher frequencies compared to that of the unmodified PP. By adapting the process temperature to 240 °C, the shear thinning effect for PODIC and LP is even more pronounced. Particularly, PODIC at higher temperature shows an increasing complex viscosity compared to that of the unmodified PP. Moreover, the shear thinning effect was significantly more distinct with respect to the other samples. A higher viscosity compared to that of the virgin PP and a higher shear thinning effect at higher frequencies are an indication of a larger amount of LCB, which could be generated during the reactive extrusion at 240 °C.

In addition, the trend of the viscosity curves shows nearly the same trend as the MFR values. For example, the viscosity curves of BEC display the lowest viscosity level at both extrusion temperatures (180 and 240 °C), and therefore the highest MFR values.

**Figure 5:**
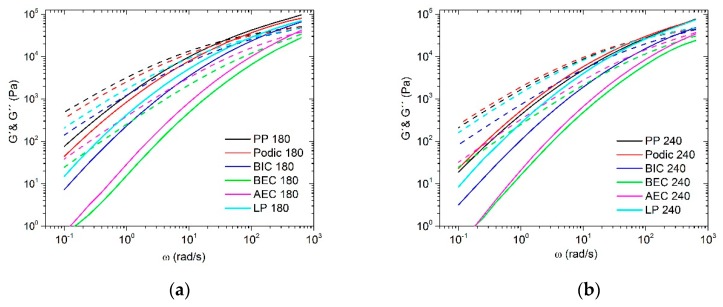
Storage and loss moduli as a function of angular frequency of samples extruded at (**a**) 180 °C; (**b**) 240 °C; measured at 230 °C.

In addition to the complex viscosity, the storage modulus G’ and loss modulus G’’ (dashed lines) play an important role in the dynamic rheology. Both parameters provide information about viscous and elastic effects and the influence of shear rate or frequency on material functions [50]. The curves of G’ and G’’ of 180 and 240 °C extruded materials are presented in Figure 5. In all cases except for PODIC at 240 °C, it can be observed that the curves i.e., the crossover point of the modified PP, tend to have higher frequencies. Furthermore, it can be seen that not each curve has a crossover point. In particular, BEC displays no crossover points in both cases, which is attributable to the high MFR values. Morshedian et al. [51] reported that as the MFR increases, the crossover point shifts to higher frequencies. 

In the literature, it is well established that changes in molecular mass *M*_W_ and molecular mass distribution (MMD) of different polymers can be detected by the position change of the crossover point [51,52,53,54]. This means that there is a critical frequency ω_C_ and a critical modulus G_C_ for each crossover point. The vertical shift of G_C_ provides information about the MMD, which means that an increasing G_C_ indicates a narrower MMD. The change in *M*_W_ can be determined with the shift of ω_C_. The horizontal shift towards higher frequencies indicates a decrease in *M*_w_ [55]. 

The values of ω_C_ and G_C_ of the unmodified and modified samples are presented in Table 3. At 180 °C, the modification with the peroxides leads to a lower molecular mass, which is represented by higher ω_C_ values compared to those of the unmodified PP. Moreover, the MMD is lower in comparison to the unmodified PP, caused by the higher G_C_ values. The modification at 240 °C shows almost the same results regarding ω_C_ and G_C_. For BEC and AEC, a crossover could not be obtained for the 240 °C extrusion temperature in the investigated angular frequency range. No data supported statement can therefore be made, but it can be assumed that a possible crossover point would occur at a frequency higher than 628 rad/s. The degradation of PP using these peroxides is very strong. Only PODIC presented a higher MW and MMD compared to PP at 240 °C, which indicates that LCB dominated in this case.

**Figure 6:**
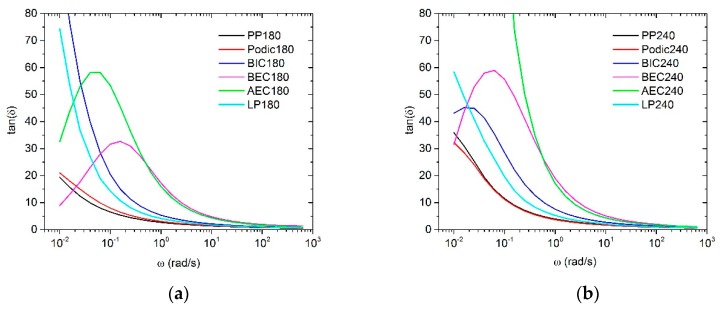
tanδ of the unmodified and modified PP with extrusion temperature of (**a**) 180 °C and (**b**) 240 °C; measured at 230 °C.

According to Wood-Adams et al. [56], the presence of LCB can be evaluated by using the plot of the loss angle δ and tanδ as a function of the frequency ω. Furthermore, De Maio and Dong [57] reported that higher elasticity leads to higher melt strength, which means that melt strength and tanδ are related to each other. The curves of all samples are shown in Figure 6. Normally, the linear polymer melt represents a monotonic decrease in tanδ by increasing ω. This behavior demonstrates the unmodified PP and the modified PP with PODIC at 180 °C. Their curves have nearly the same trend; only the gradient of PP is more pronounced. By comparison, BIC and LP show a typical terminal behavior of liquid-like materials because of the rapid decrease of tanδ with the increase of ω. BEC and AEC have an unexpected curve progression, which is not usual for conventional PP. Li et al. [48] and Sugimoto et al. [58] reported a similar unexpected rheological behavior of tanδ for their investigated samples. It is known in the literature that binary blends show a two-step rubbery plateau, which can only be seen for high molecular weight components [59,60,61,62]. However, the modification at 240 °C presented nearly the same behavior, whereas BIC also showed a second plateau and the curves of all samples displayed a steeper curve progression compared to that of the modification at 180 °C. The formation of such an unexpected curve progression supports the hypothesis of a strong degradation when using AEC, BEC, and BIC (at a higher temperature). All of them presented high MFR values, the lowest viscosity curves, and crossover points at high frequencies, or even no crossover points, which indicates that a possible degradation (β-scission) of PP is predominant.

### 3.3. Molar Mass Determination

In addition to determining the MMD using dynamic rheology, high-temperature gel permeation chromatography (HT-GPC) measurements were performed. GPC is one of the most important methods to determine the MMD of polymers [63]. GPC measurement, a well-established concept in the literature, provides a further possibility to determine the molecular structure and any modifications made to polymers [51,64,65,66,67,68].

**Figure 7:**
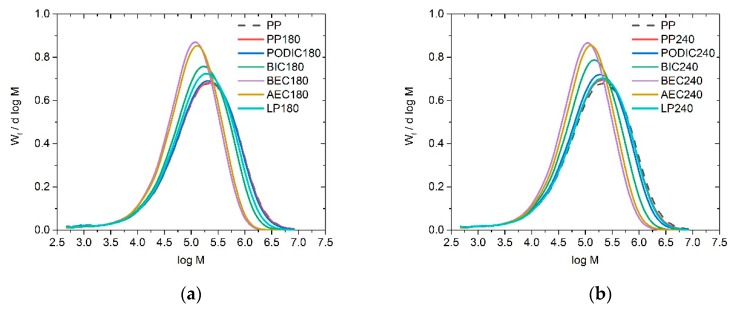
MMD of virgin PP as well as unmodified and modified PP samples at (**a**) 180 °C and (**b**) 240 °C.

The corresponding curves of the GPC measurements are illustrated in Figure 7. The MMD of the virgin PP (without extrusion) was used for both cases as reference (dashed lines). In Figure 7a, the curves present nearly the same behavior compared to those of the reference PP and the unmodified PP. Only AEC and BEC show a significant change in MMD curves. The distribution becomes narrower and the peak maximum of MMD is distinctively increased and shifts to lower M values. The distribution of all samples, which were extruded at 240 °C, and the reference PP are shown in Figure 7b. It can be observed that all curves have nearly the same trend as the MMD curves at 180 °C. Therefore, the temperature difference during extrusion (180 °C or 240 °C) has no influence on the MMD via GPC. Only the use of the different peroxides presented a change in the distribution. 

Additionally, the GPC results are listed in Table 4. PP and PODIC (both extruded at 180 °C) display almost similar molecular mass with only slight differences in their distributions. With the addition of the other peroxides, *M*_w_ and *M*_w_/*M*_n_ decreased in all systems, especially BEC and AEC, which indicates the occurrence of degradation. The modification at 240 °C presented slightly the same trends for the molecular mass and polydispersity compared to those of the modification at 180 °C. Only LP 240 shows a lower degradation in comparison to LP 180. Therefore, PP 240, PODIC 240, and LP 240 display similar values, which means that LCB and degradation occur concurrently.

### 3.4. Extensional Rheology

For a better verification of LCB, additional extensional rheology measurements were performed on the different formulations. According to Münstedt et al. [69], the extensional behavior of a material is very important for many processing operations such as thermoforming, blow molding, film blowing, film casting, foaming, and so on. 

In previous work performed by our group, some polymers, especially PP-LCB, have a pronounced increase of extensional viscosity compared to their virgin linear materials [32,70]. This phenomenon is called strain hardening, which is strongly related to LCB and the nascent changes in the molecular structures of PP [28,29].

**Figure 8:**
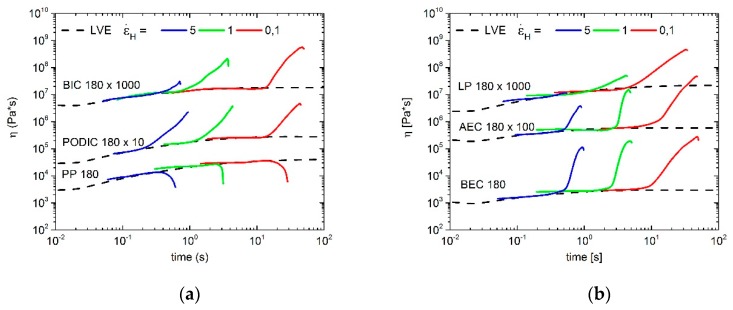
Extensional rheology of the different PP samples, (**a**) unmodified PP, PODIC and BIC; (**b**) BEC, AEC and LP, extruded at 180 °C; measured at 180 °C.

As shown in Figure 8, the linear PP at 180 °C does not exhibit a strain hardening behavior and therefore there is no remarkable deviation from the linear viscoelastic curve (LVE). The LVE confirms an increase that is three times the shear viscosity [71]. In addition to the previously mentioned linear PP, extensional rheology measurements of all modified PP samples demonstrate a strain hardening behavior at three different Hencky strain rates at 180 °C. LP does not display a deviation from LVE and therefore no strain hardening threat at the highest Hencky rate. However, all in all, the modification with the different peroxides and the LCB induced by reactive extrusion at 180 °C were successfully obtained. 

**Figure 9:**
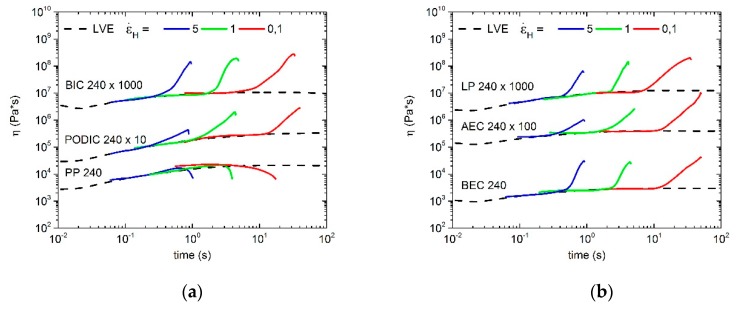
Extensional rheology of the different PP samples, (**a**) unmodified PP, PODIC and BIC; (**b**) BEC, AEC and LP, extruded at 240 °C; measured at 180 °C.

The extensional rheology curves of all PP-LCB extruded at 240 °C are plotted in Figure 9. As expected, the linear PP at this temperature does not show any strain hardening, whereas all of the modified PP samples have a strong increase of the extensional rheology curves. This means that long-chain branching of PP is possible at temperatures higher than 180 °C, which is very important for different applications in the industry. 

With the strain hardening coefficient SH, it is possible to quantify the strain hardening behavior of each sample according to Equation (3), where η_e_ (t) is the maximum extensional viscosity of the Hencky strain rate and η_e_^0^ (t) is the extensional viscosity of the LVE curve.

SH = η_e_ (t)/η_e_^0^ (t) (3)

**Figure 10:**
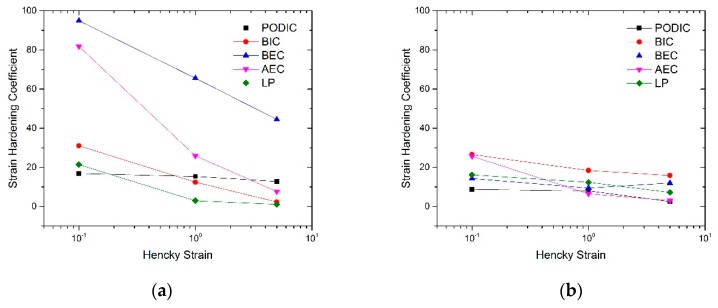
Strain hardening coefficient at different strains rates (0.1, 1, 5); modified at (**a**) 180 °C and (**b**) 240 °C.

The calculated strain hardening ratios of all samples are given in Figure 10. The modification at 180 °C for BEC and AEC achieve very high strain hardening values at a Hencky strain of 0.1. However, the SH factor decreases significantly by increasing the Hencky strain for both peroxides. A similar curve progression displays BIC and LP at 180 °C. Only PODIC shows a constant behavior. In comparison, the modification at 240 °C shows lower strain hardening coefficients for all mixtures and more linearity in the logarithmic plot. 

### 3.5. Mechanical Properties

**Figure 11:**
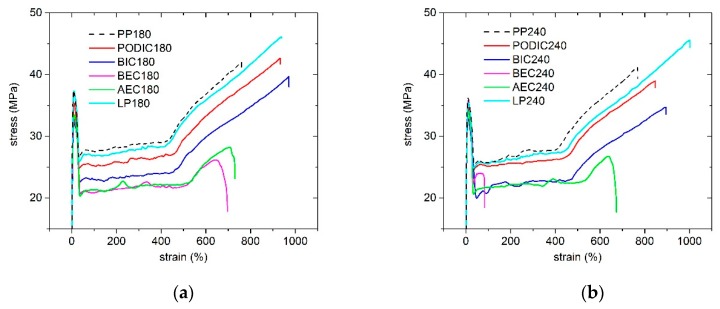
Stress–strain plots of unmodified PP and modified PP extruded at (**a**) 180 °C and (**b**) 240 °C.

**Figure 12:**
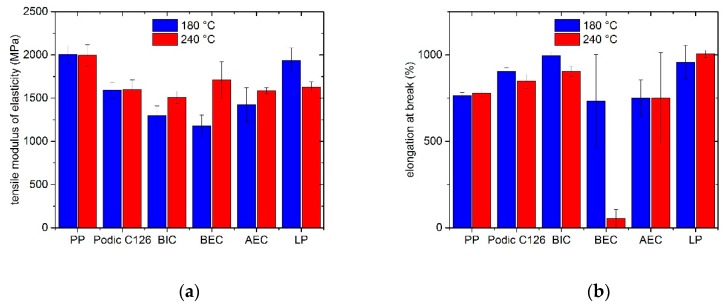
Results of the tensile test: (**a**) tensile modulus of elasticity and (**b**) elongation at break of the model mixtures extruded at 180 °C and 240 °C.

The tensile test plays an important role in determining the mechanical properties of polymers such as tensile strength, yield point, and elongation at break [72,73,74,75]. In Figure 11, the stress–strain plots at extrusion temperatures of 180 and 240 °C are shown, and the obtained results of tensile modulus of elasticity and elongation at break are presented in Figure 12. For the stress–strain plots, exemplary curves of each sample are illustrated. The stress–strain curves at the 180 °C extrusion temperature show consistently high strain values, which is an indication that cross-linked networks do not exist [76]. Furthermore, it can be seen that the yield point decreases significantly when using BIC, BEC, and AEC for the modification, whereas the yield point of PODIC decreases slightly and LP shows the same value compared to PP 180. In particular, the modification with PODIC, BIC, and LP displays a well-improved strain hardening behavior, which could be the result of the entanglement from introducing LCB. The different levels of the curves suggest that the degradation of PP-LCB could play a role, because BIC, BEC, and AEC illustrate the lowest levels of the curves. 

All in all, the stress–strain plot at the 240 °C extrusion temperature presents the same results as those of the plot at 180 °C, except that BEC 240 °C has a very brittle behavior compared to the other curves at 240 °C and also to the stress–strain curve of BEC 180 °C. It is also apparent that the strain hardening of LP 240 is more significantly pronounced compared to that of PODIC 240 and BIC 240.

On the one hand, the tensile modulus of elasticity of unmodified and modified PP and, on the other hand, the elongation at break are displayed in Figure 12. After the reactive extrusion, the tensile modulus of elasticity for the unmodified PP is nearly constant. After adding the different peroxides, the tensile modulus of elasticity decreases significantly. Kamleitner et al. [30] reported a similar behavior for modifications with PODIC and suggested this phenomenon with the formation of LCB and long-chain aliphatic decomposition products from the peroxide. It is shown that all modified formulations at 240 °C have consistent values of tensile modulus of elasticity. Compared to these, the values at 180 °C vary slightly more depending on which peroxide is used. However, by using LP as peroxide for the reactive extrusion, the highest tensile modulus of elasticity values can be achieved. 

The elongation at break can be increased or held constant by adding peroxide. The single exception is shown for the formulation with BEC at 240 °C. In this case, the elongation at break was close to zero and all samples show a brittle behavior. However, the peroxides PODIC and BIC obtained higher values and, therefore, the LCB of PP has a strong effect on the tensile strength. Specifically, the modification with LP shows the highest elongation at break at both temperatures. It was possible to increase the values by about 30% compared to the unmodified PP samples.

**Figure 13:**
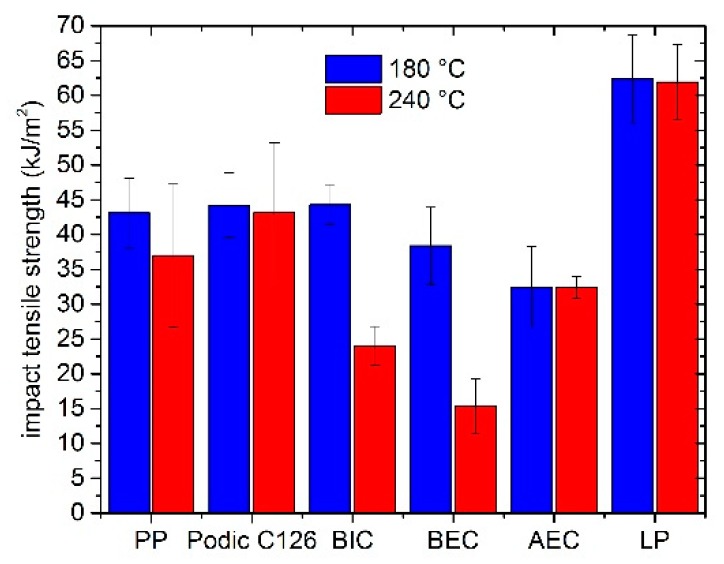
Results of the impact tensile test of the different model mixtures prepared at 180 °C and 240 °C.

The results of tensile impact tests are shown in Figure 13. At 180 °C, the unmodified PP and the samples with PODIC and BIC have nearly the same impact tensile strength. Compared to this, BEC and AEC show a slight downward trend. Interestingly, the impact tensile strength of LP increased to 62 kJ/m^2^, which conforms to 50% more than unmodified PP.

Additionally, the reactive extrusion at 240 °C shows that PODIC can maintain the same level of tensile impact strength as at 180 °C. In comparison, the unmodified PP loses a little of its impact strength after extruding at 240 °C. By adding AEC, BIC, or BEC, the impact strength decreases significantly. However, by adding LP, a strong enhancement can be achieved. The impact strength can be maintained at the same level as LP at 180 °C and, compared to unmodified PP, the modification with LP at 240 °C raises the value by about 70%.

## 4. Conclusions

In this study, the effects of different peroxide types (symmetrical and unsymmetrical structure) on the LCB behavior of PP was investigated. The PP-LCB samples were prepared via reactive extrusion in melt by temperatures of 180 °C and 240 °C. As expected, it was found that, for the virgin PP, the temperature of the extrusion process plays an important role regarding its melt behavior. After increasing the extrusion temperature to 240 °C, the MFR value was higher and the complex viscosity was lower in comparison to the values at 180 °C. 

The modification with peroxides showed different results. It was demonstrated that the reactive extrusion with unsymmetrical peroxides (BIC, BEC, and AEC) led to higher MFR values and showed lower complex viscosity curves. In comparison, the use of symmetrical peroxides (PODIC and LP) showed small deviations from the virgin PP, which means that the application of these two peroxides is more stable. This trend was better depicted by applying frequency sweep and performing GPC measurements. It can be concluded that the unsymmetrical peroxides did not show a crossover point in the suggested frequency area in comparison to all other samples. The curves shifted to higher frequencies, which means that the values of MW and MMD were lower compared to those of the unmodified PP. This was the case for BEC and AEC at the 180 °C extrusion temperature and for BIC, BEC, and AEC extruded at 240 °C. Additionally, the GPC measurements showed for BEC 180, AEC 180, and BIC 240 a shift of the MMD peak maximum to lower M values, i.e., a narrower progression of MMD. These results suggested that the degradation with unsymmetrical peroxides was more fundamental compared to symmetrical peroxides. Furthermore, these measurements allowed us to conclude that PODIC and LP provide the best opportunities to generate LCB-PP. 

The strain hardening behavior was measured using extensional rheology. It was demonstrated that, for all peroxides, an improvement in strain hardening as well as in melt strength was achieved. This knowledge and the previously discussed results show the competition between degradation and LCB reactions. 

Furthermore, it was shown that the mechanical properties of the samples modified by using peroxides are very dependent on the extrusion temperature. The elongation at break values of the samples that were extruded at 180 °C showed that, on the one hand, the modification with peroxides could be held constant in the case of BEC and AEC compared to PP 180 and that, on the other hand, it could be improved by using PODIC, BIC, and LP. In comparison to PP 180, the modification resulted in an improvement, that is, in the case of PODIC nearly 20%, for BIC 30%, and for LP 25%. At the extrusion temperature of 240 °C, the improvements were not so significant with the peroxides. However, in comparison to PP 240, the elongation at break values could be increased with PODIC by nearly 10%, with BIC by about 15%, and with LP by nearly 30%. For the tensile impact test, the modification with the unsymmetrical peroxides (BIC, BEC, and AEC) had the tendency to lower impact tensile strength values, which is an indication of chain scission and the related degradation, compared to the unmodified PP at both extrusion temperatures [25,77,78]. Nevertheless, the modification with PODIC showed in both cases a constant level in comparison to the unmodified PP. In particular, the use of LP improved the impact tensile strength. At 180 °C, an improvement of about 40% was achieved, and for the extruded samples at 240 °C, the improvement increased to about 60% compared to PP 240.

All in all, it can be concluded that the modification of PP with PODIC and LP (symmetrical peroxides) offers the best possibilities to generate PP-LCB at an extrusion temperature of 180 °C and also 240 °C. In both cases, an improvement of their properties can be achieved, such as higher elongation at break values or higher impact tensile strength and the availability of strain hardening. 

These results are very promising in view of possible “upcycling” options to recycle individual PP flows in commercialized products. Furthermore, reactive extrusion at higher temperatures—which are common in industrial practices—was successfully performed.

## Figures and Tables

**Table 1:** Characteristics of the peroxides.

Peroxide	CAS-No.:	Max. Storage Temperature (°C)	Molecular Weight (g/mol)	Active Oxygen Assay (%)	10 h Half-Life Temperature (°C)
PODIC C126 *	53220-22-7	15	514.8	2.95	48
LP *	105-74-8	30	398.6	3.97	61
BIC **	2372-21-6	25	176.2	6.81	98
BEC **	34443-12-4	30	246.3	6.17	98
AEC **	70833-40-8	25	260.4	5.78	95

* symmetrical peroxide; ** unsymmetrical peroxide.

**Table 2:** Sample compositions and processing temperatures.

Sample	Extrusion Temperature (°C)	Sample Specification
PP 180/PP 240	180/240	Virgin PP granulate
PODIC 180/PODIC 240	180/240	20 mmol/kg PODIC C126 (1 wt %)
BIC 180/BIC 240	180/240	20 mmol/kg BIC (0.5 wt %)
BEC 180/BEC 240	180/240	20 mmol/kg BEC (0.5 wt %)
AEC 180/AEC 240	180/240	20 mmol/kg AEC (0.5 wt %)
LP 180/LP 240	180/240	20 mmol/kg LP (0.8 wt %)

**Table 3:** Summary of the rheological data from the frequency sweep measured at 230 °C.

Sample	ω_C_ (rad/s)	G_C_ (kPa)	Comment
PP 180	36	23.9	
PODIC 180	50	24.5	MW ↓, MMD ↓
BIC 180	139	29.0	MW ↓, MMD ↓
BEC 180	-	-	(MW ↓, MMD ↓) *
AEC 180	460	33.6	MW ↓, MMD ↓
LP 180	84	25.6	MW ↓, MMD ↓
PP 240	98	27.1	
PODIC 240	66	23.5	MW ↑, MMD ↑
BIC 240	308	32.6	MW ↓, MMD ↓
BEC 240	-	-	(MW ↓, MMD ↓) *
AEC 240	-	-	(MW ↓, MMD ↓) *
LP 240	114	28.4	MW ↓, MMD ↓

* no crossover point detected but estimated from the general trend of the curves.

**Table 4:** Summary of the GPC data; molar mass and polydispersity of all extruded samples at 180 °C or 240 °C.

Sample	*M*_w_ (kg mol^−1^)	*M*_n_ (kg mol^−1^)	*M*_w_/*M*_n_
PP 180	379	63.2	6.0
PODIC 180	359	62.8	5.7
BIC 180	263	57.4	4.6
BEC 180	164	48.3	3.4
AEC 180	179	50.1	3.6
LP 180	307	59.9	5.1
PP 240	354	62.2	5.7
PODIC 240	309	60.5	5.1
BIC 240	230	54.1	4.2
BEC 240	160	46.8	3.4
AEC 240	179	50.0	3.6
LP 240	341	62.0	5.5
